# Analysis of the Open Die Forging Process of the AZ91 Magnesium Alloy

**DOI:** 10.3390/ma13173873

**Published:** 2020-09-02

**Authors:** Grzegorz Banaszek, Teresa Bajor, Anna Kawałek, Tomasz Garstka

**Affiliations:** Faculty of Production Engineering and MATERIALS Technology, Czestochowa University of Technology, 42-201 Czestochowa, Poland; grzegorz.banaszek@pcz.pl (G.B.); anna.kawalek@pcz.pl (A.K.); tomasz.garstka@pcz.pl (T.G.)

**Keywords:** magnesium alloy AZ91, physical modelling, open die forging, flat anvils, shaped anvils

## Abstract

The paper presents the results of numerical modelling of the forging process of magnesium alloy ingots on a hydraulic press with the use of flat and shaped anvils. The use of shaped (*rhombic-trapezoid*) anvils will affect the uniform distribution of temperature and strain intensity in the entire volume of the forging, causing a number of forging passes, which in consequence will reduce the costs of the blank manufacturing process. However, higher values of the strain intensity were obtained during the deformation of the material in flat anvils. The purpose of the research was to propose assumptions for forging technology of producing a blank from AZ91 alloy with the use of flat and shaped anvils. Numerical examination for AZ91 magnesium alloy was carried out using the Forge^®^NxT commercial software. The rheological properties of the investigated alloy were determined on the basis of uniaxial compression tests carried out in the Gleeble 3800 metallurgical simulation system. The numerical analysis of the process of forging AZ91 alloy ingots on a press was conducted in the temperature range of 200–400 °C and at several forging passes.

## 1. Introduction

The unabated search for lightweight structural materials characterized by favourable strength properties in recent years results in increased interest in magnesium alloys [1,2,3,4,5,6,7,8,9,10]. Owing to their properties, alloy-derived products are attractive for many trades, which include the automotive, shipbuilding, aviation, aerospace and electronics industries [11,12,13]. These alloys exhibit the highest strength relative to density, which favourably affects their application in the production of lightweight structures. The majority of magnesium alloy semifinished products, used to manufacture, among others, structural elements, are produced using casting methods. Most frequently, magnesium alloys are formed within the extrusion processes, and less often within rolling or forging. Furthermore, owing to the reliability, functionality and mechanical properties, forged magnesium alloy parts are used as structural elements with stringent requirements. These features are of particular importance in transport, which makes this industry the main buyer of forged parts. Due to the fact that the open die forging processes enables free shaping of the metal flow kinematics, among others, through controlling the shape and dimension parameters of the die working surfaces and the main parameters of the elongation operation, which is largely limited and sometimes even impossible in the case of extrusion processes involving the discussed magnesium alloys. Conducting studies in the field of forging technology engineering requires a comprehensive approach to the proposed research problem. The high variability of shaping parameters (temperature, order of material rotation and die impacts during forging elongation reduction values, relative stroke values, strain rate, and the shape and dimension parameters of die working surfaces) makes it extremely difficult to obtain high-quality magnesium alloy forgings with a uniform microstructure throughout the entire volume. Due to the hexagonally compact crystallographic structure, magnesium alloys exhibit limited plasticity and poor formability at ambient temperatures. They are also characterized by high mechanical anisotropy arising in the course of plastic deformation. These factors result in an observed increase of the demand for the search for a production technology providing magnesium alloys with better strength properties. The correct implementation of the open die forging process in flat and shaped dies involving selected magnesium alloys requires not only special forging equipment, but most of all, necessary specialist knowledge in this field. The AZ91 alloy will undoubtedly find application in its forged form, since it reaches higher strength than the AZ31 and AZ61 alloys, which is the result of Al content. The problematicMg_17_Al_12_ binary phase, usually found at grain boundaries, can be fully dissolved through appropriate thermal treatment, which should be used to precipitate hardening of the alloy. The use of numerical research with the finite element method in the design of engineering structures is very important from the point of view of the economics process as well as the design and implementation of the technology of manufacturing semifinished products [14,15]. Undertaking research on the numerical modelling of AZ91 magnesium alloy plastic forming is one of the initial stages in the development of a technology for manufacturing finished goods [16,17,18,19].

## 2. Test Objective and Scope

The objective of the study was to determine temperature distributions and strain fields in AZ91 magnesium alloy bars elongated with the open die forging method, in flat and shaped dies, based on the model tests of these technological processes.

Forging was modelled using the Forge^®^NxT commercial software (version 2.0) based on the FEM, which enables tracking temperature and strain intensity changes in metal during plastic forming, at any forging stage. The analysis of numerical test results will be used as a base to develop guidelines for the production of magnesium alloys bars using the open die forging method, in flat and shaped dies. The test results should contribute to improving the structural and mechanical properties of AZ91 magnesium alloy bars through the appropriate selection of the process parameters for open die forging in flat and shaped dies.

Plastometric tests were also conducted and used as a base to develop stress-true strain relationship graphs for the AZ91 alloy and to match flow stress function coefficients, which were used in the numerical study.

The strain, strain rate and temperature ranges were adopted during the theoretical studies based on the characteristics of the machines used within real forging process and source literature data [18,20].

## 3. Materials and Methods

The material chosen for the tests was magnesium alloy AZ91 with chemical compositions as given in Table 1.

## 4. Determining Rheological Properties of a Selected Magnesium Alloy

The basic feature characterizing the plastic formability of material in plastic working processes is the flow stress *σ**_p_*** and limit strain $\varepsilon_{g}$.

The flow stress *σ_p_*, which is the stress necessary to initiate and continue the metal plastic flow in uniaxial stress conditions is a function of strain (*ε*), strain rate ($\overset{•}{\varepsilon }$), temperature (*T*) and strain course history.

Determining the process plasticity characteristics is particularly difficult in hot plastic working conditions, since the material structure simultaneously experiences processes arising from the plastic strain mechanism, as well as strengthening processes, heat activated processes and processes depending on the phenomenon duration, leading to material weakening.

Within the available software intended for solving problems in the field of metal plastic flow or for calculating the strain forces and powers using the finite element method, the flow stress *σ**_p_*** values are determined based on the adopted flow stress function. Most often, flow stress is expressed by a relationship in the form of $\sigma_{p}=(\varepsilon ,\overset{•}{\varepsilon },T)$. Numerous functions are used for the mathematical description of the *σ**_p_*** values, depending on the strain *ε*, temperature *T* and strain rate $\overset{•}{\varepsilon }$. One of them is the Hansel-Spittel Equation (1) [21]. This relationship is frequently used to determine the *σ**_p_*** value in software for the numerical modelling of plastic working processes [22]:

(1) $$\sigma_{p}=Ae^{m_{1}T}\varepsilon^{m_{2}}{\dot{\varepsilon }}^{m_{3}}\varepsilon^{\frac{m_{4}}{\varepsilon }}{\left(1+\varepsilon \right)}^{m_{5}T}\varepsilon^{m_{7}T}{\dot{\varepsilon }}^{m_{8}T}T^{m_{9}}$$

where: *σ**_p_*** is the flow stress, *ε* is the true strain, $\dot{\varepsilon }$ is the strain rate, *T* is the temperature, m_1_–m_9_ are coefficients characterizing magnesium alloys.

**Figure 1:**
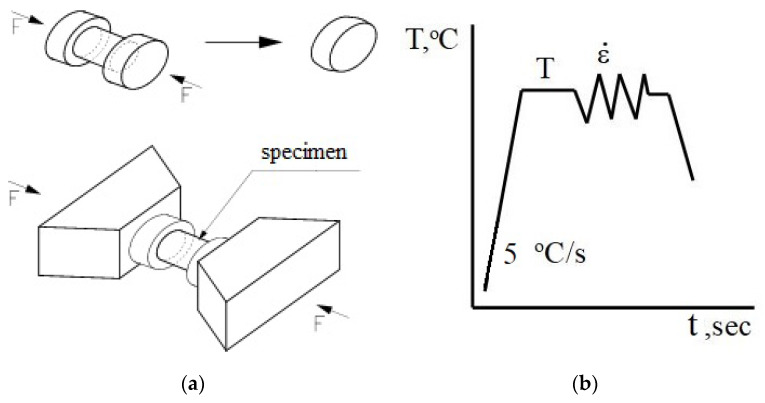
Geometry and dimension of load (**a**); and thermal cycle of physical simulation (**b**).

In the paper, the rheological properties of the AZ91 magnesium alloy were determined based on compression tests conducted with a Gleeble 3800 simulator (Dynamic System Inc., Poestenkill, NY, USA) for metallurgical processes, which is owned by the Department of Plastic Working and Safety Engineering of the Częstochowa University of Technology [23]. The Gleeble 3800 simulator enables testing over a wide temperature range, corresponding to the actual conditions within an analysed process. The tests are carried out in a vacuum chamber, at a constant temperature of the strained sample. The plastometric tests involve cylindrical samples, with a diameter of 10 mm and a length of 12 mm (Figure 1a). The uniaxial compression tests involve compression of cylindrical samples located between two well-lubricated planes. Given the ideal settings, sample compression should take place in isothermal conditions. The mutual interaction area between planes and samples should have a zero friction coefficient value. Furthermore, compressed samples should not be deformed resulting in the loss of the cylindrical shape. Taking these conditions into account and assuming the invariability of metal volume during the compression tests, the determined relationships between the true stress and the true strain should be very similar to the conditions found when forming metal in the course of industrial processes.

The advantage of an uniaxial compression test at an elevated temperature is the fact that the data on the true stress relative to the true strain can be obtained for a significantly wider strain range compared to the strains studied, e.g., by the tensile test.

The phenomenon of friction occurs in almost each case of the uniaxial compression tests, and the upsetting tools are subject to slight cooling. Using Gleeble 3800 simulator enables minimizing friction through the application of lubricants and a simultaneous reheating of the tools.

The plastometric tests were conducted with the following parameters:

- sample temperature: 200 °C, 300 °C, 400 °C,
- strain rate: 0.1 s^−1^, 1 s^−1^, 10 s^−1^,
- true strain: max. 0.8.

The samples were heated at a constant rate of 5 °C/s, up to a preset temperature, held at this temperature for 20 s and then compressed. The diagram for conducted tests is shown in Figure 1b.

**Figure 2:**
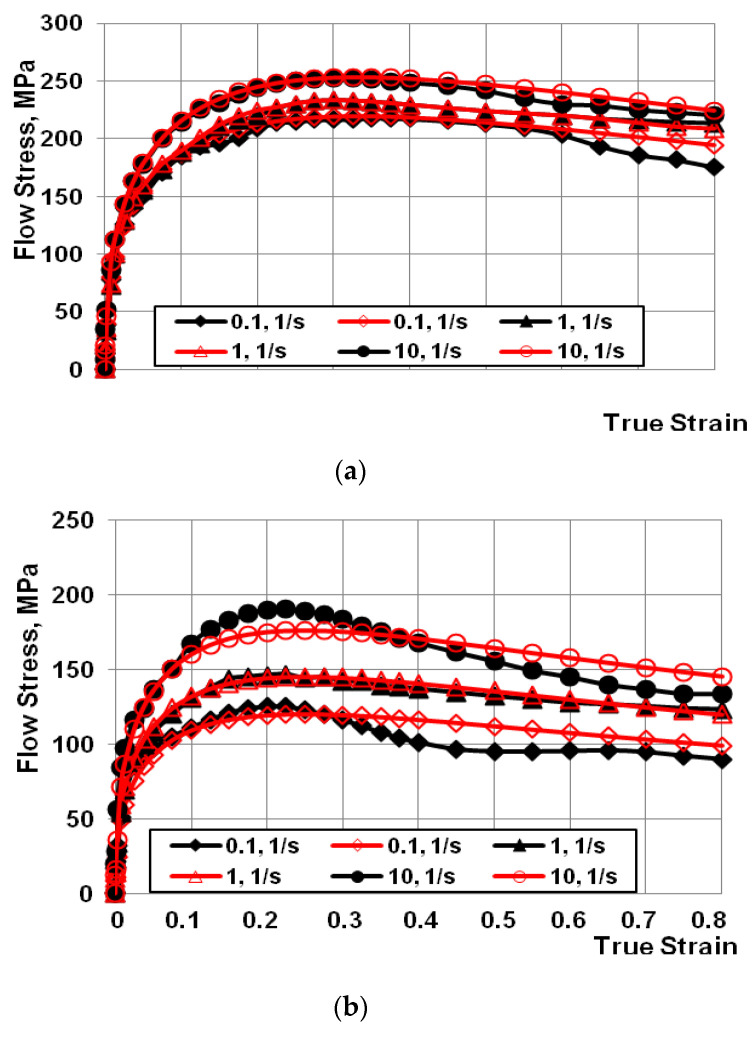

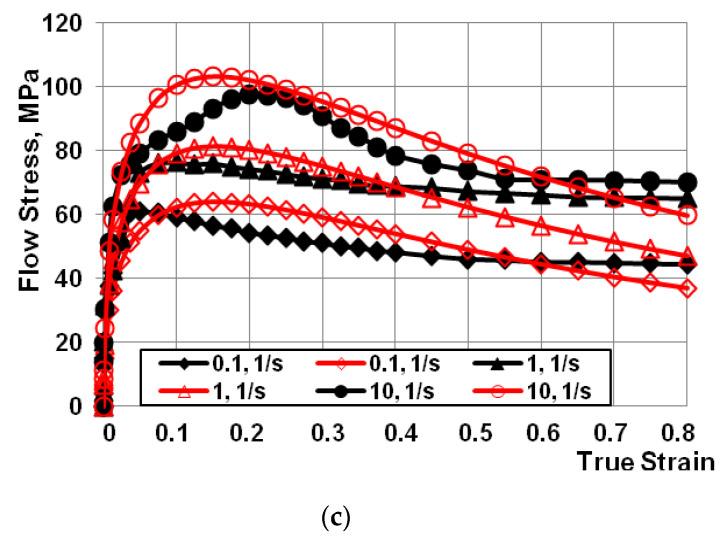
Work-hardening curves for the strain rate range of (0.1 s^−1^–10.0 s^−1^) at a temperature of: (**a**) 200 °C, (**b**) 300 °C; (**c**) 400 °C. (Black indicates the experimental curves; red indicates the approximated curves).

Graphs showing the dependence of flow stress from true strain for the AZ91 magnesium alloy were developed based on the conducted plastometric tests (Figure 2a–c). After the results of plastometric tests were approximated, the coefficients of Equation (1) were determined (Table 2).

The data showed in Figure 2 indicates that in the case of the entire studied range of sample strain rates and temperatures, after reaching the critical strain, the stress value decreases along with increasing true strain value.

## 5. Open Die Forging and Shaped Die Forging Tools

The first stage of engineering a technological forging process is the proper selection of shape and dimension parameters of the dies. The geometric shape and dimensions of the die forging tools were selected based on a literature study for the purposes of the research paper subject and previous studies conducted at the Department of Plastic Working and Safety Engineering of the Czestochowa University of Technology [24,25,26,27,28,29,30,31,32,33].

**Figure 3:**
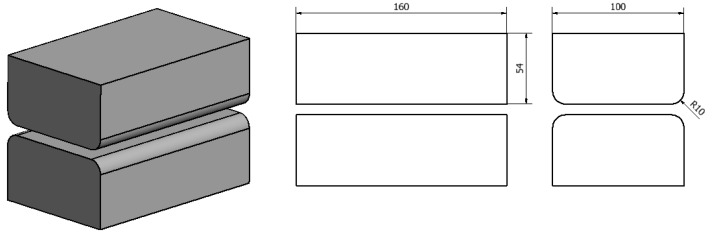
Shape and dimensions of flat dies used for AZ91 magnesium alloy forging.

**Figure 4:**
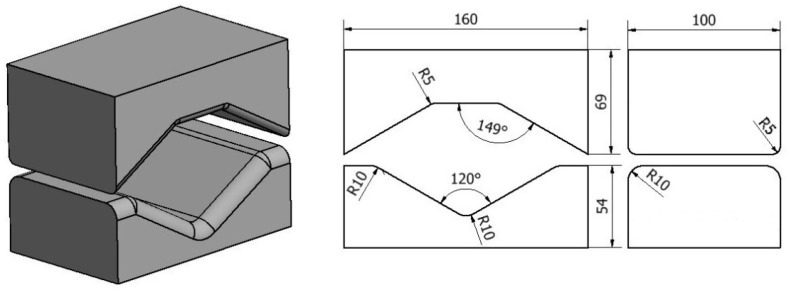
Shape and dimensions of rhombic-trapezoid dies used for AZ91 magnesium alloy forging.

Shapes and dimensions of dies used for the theoretical tests of the forging process are shown in Figure 3 and Figure 4.

**Figure 5:**
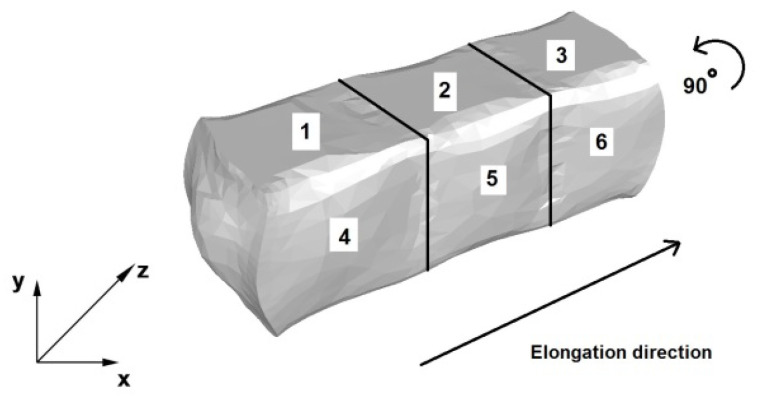
Flat die elongation process diagram.

**Figure 6:**
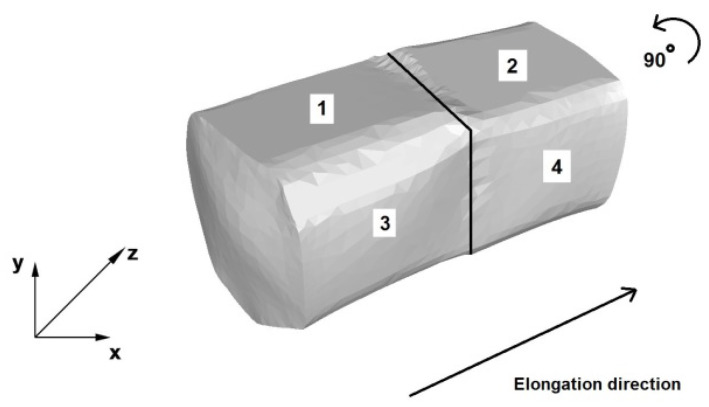
Trapezoid-rhombic die elongation operation diagram.

Main process parameters of the forging operation were selected based on our previous research and source literature [24,25,26,27,28,29,30,31,32,33]. The upper anvil speed was adopted at V = 8 mm/s, while the lower anvil was stationary. The description and flow diagram of the elongation operation are shown in Figure 5 and Figure 6.

Figure 5 contains a diagram of elongation an AZ91 alloy bar in flat dies. The first stage, three single-sided forging passes with a forging reduction of 40% and a relative feed of 0.8 were made, as per the forging direction indicated in the figure. Next, the forging was rotated, as shown in Figure 5 and the dies returned to their starting position. This was followed by three consecutive forging passes (4, 5 and 6), with the same forging reduction and relative feed.

Figure 6 shows the diagram of an AZ91 alloy bar elongation operation in trapezoid-rhombic dies. The first stage of the process involved two first forging passes, with single-sided forging, relative forging reductions of 25% and relative feed of 0.8. Next, the forging was rotated, as shown in Figure 6, the dies returned to the start of the forging, in the place of the Cartesian coordinate system, and two subsequent forging passes (3 and 4) were conducted, with a forging reduction of 40% and the same relative feed.

## 6. Determination of Boundary Conditions for the Magnesium Alloy Forging Process

Forge^®^NxT software, which allows for the thermo-mechanical simulation of plastic working processes, was selected for the numerical modelling of the AZ91 magnesium alloy forging process. A detailed description of the temperature, power, and stress and strain functionalities, as well as the thermo-mechanical and friction laws used for the calculations can be found in the research paper [34]. The Galerkin equation embedded in the software was used for the thermal calculations within the analysis [34], whereas hardening curves were approximated using the Henzel-Spittel Equation (1).

The simulations for the purposes of the research paper utilized a thermo-viscoplastic model of a deformed body, based on the theory of high plastic strains.

The simulation adopted a cylindrical reference batch with a square section of 80 mm × 80 mm and a length *l* = 80 mm. The number of nodes adopted for the simulation equalled 9116, while the number of tetrahedral finite elements was 41,568.

The friction coefficient value adopted pursuant to Coulomb’s law was μ = 0.3.

The assumed coefficient of heat transfer between the dies and material amounted to λ_1_ = 10,000 W/m^2^K, while the coefficient of heat transfer between the metal and the environment was λ_2_ = 10 W/m^2^K. The study adopted an ambient temperature of 25 °C and a die temperature of 25 °C. The initial batch temperature before the elongation, throughout its entire volume was adopted as uniform and equal to 400 °C [35].

## 7. Analysis of the Numerical Test Results for the AZ91 Magnesium Alloy Forging in Flat and Shaped Dies

**Figure 7:**
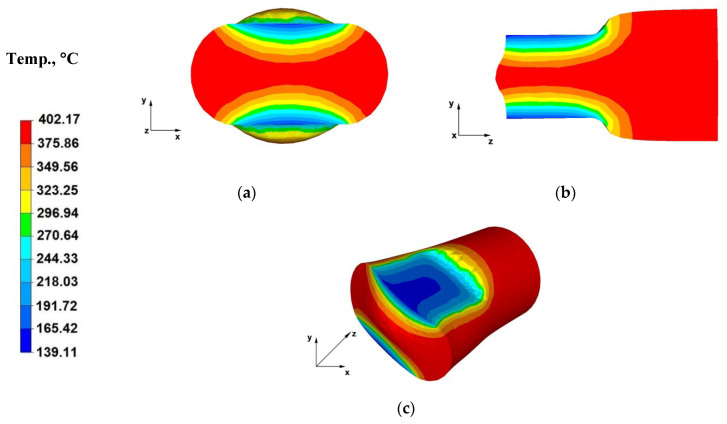
(**a**) Temperature distribution over the cross-section of an AZ91 alloy forging deformed within the first pass, with a relative reduction of 40%, in flat dies; (**b**) Temperature distribution over the longitudinal section of an AZ91 alloy forging deformed within the first pass, with a relative reduction of 40%, in flat dies; (**c**) Axonometric view of the temperature distribution over the surface of an AZ91 alloy forging deformed within the first forging pass, with a relative reduction of 40%, in flat dies.

**Figure 8:**
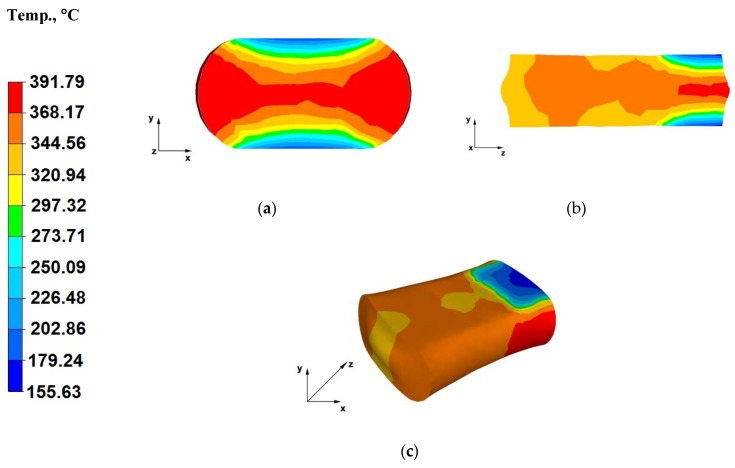
(**a**) Temperature distribution over the cross-section of an AZ91 alloy forging deformed within the third pass, with a relative reduction of 40%, in flat dies; (**b**) Temperature distribution over the longitudinal section of an AZ91 alloy forging deformed within the third pass, with a relative reduction of 40%, in flat dies; (**c**) Axonometric view of the temperature distribution over the surface of an AZ91 alloy forged within the third forging pass, with a relative reduction of 40%, in flat dies.

**Figure 9:**
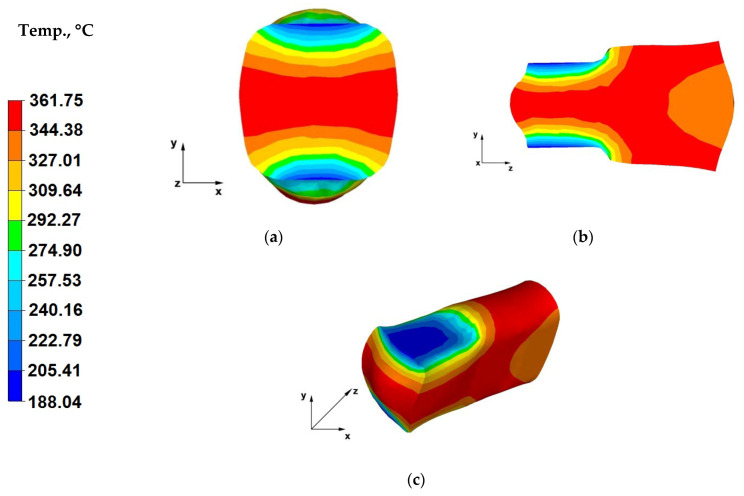
(**a**) Temperature distribution over the cross-section of an AZ91 alloy forging deformed within the fourth pass, with a relative reduction of 40%, in flat dies, after a 90° turning of the forging; (**b**) Temperature distribution over the longitudinal section of an AZ91 alloy forging deformed within the fourth pass, with a relative reduction of 40%, in flat dies, after a 90° turning of the forging; (**c**) Axonometric view of the temperature distribution over the surface of an AZ91 alloy forged within the fourth forging pass, with a relative reduction of 40%, in flat dies, after a 90° turning of the forging.

**Figure 10:**
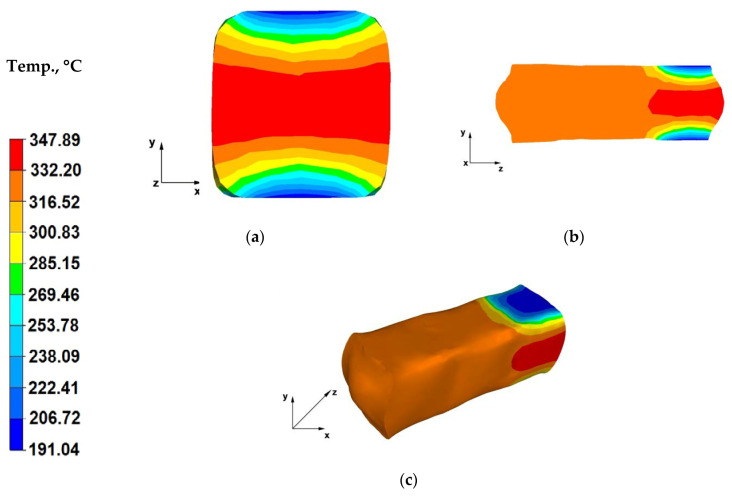
(**a**) Temperature distribution over the cross-section of an AZ91 alloy forging deformed within the sixth pass, with a relative reduction of 40%, in flat dies, after a 90° turning of the forging; (**b**) Temperature distribution over the longitudinal section of an AZ91 alloy forging deformed within the sixth pass, with a relative reduction of 40%, in flat dies, after a 90° turning of the forging; (**c**) Axonometric view of the temperature distribution over the surface of an AZ91 alloy forged within the sixth forging pass, with a relative reduction of 40%, in flat dies, after a 90° turning of the forging.

**Figure 11:**
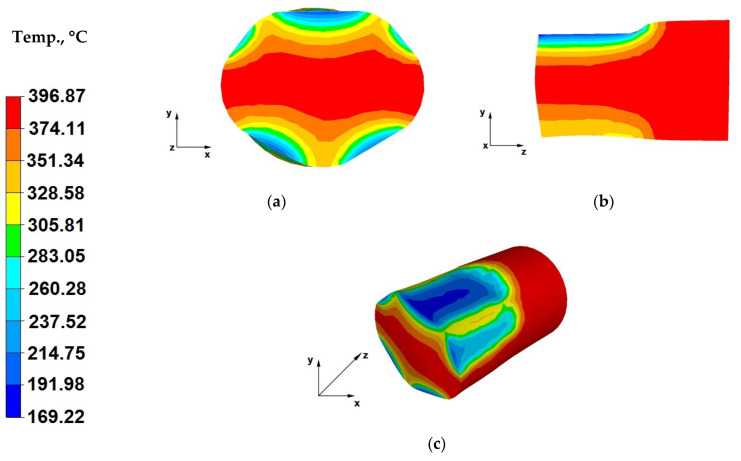
(**a**) Temperature distribution over the cross-section of an AZ91 alloy forging deformed within the first pass, with a relative reduction of 25%, in trapezoid-rhombic dies; (**b**) Temperature distribution over the longitudinal section of an AZ91 alloy forging deformed within the first pass, with a relative reduction of 25%, in trapezoid-rhombic dies; (**c**) Axonometric view of the temperature distribution over the surface of an AZ91 alloy forged within the first forging pass, with a relative reduction of 25%, in trapezoid-rhombic dies.

**Figure 12:**
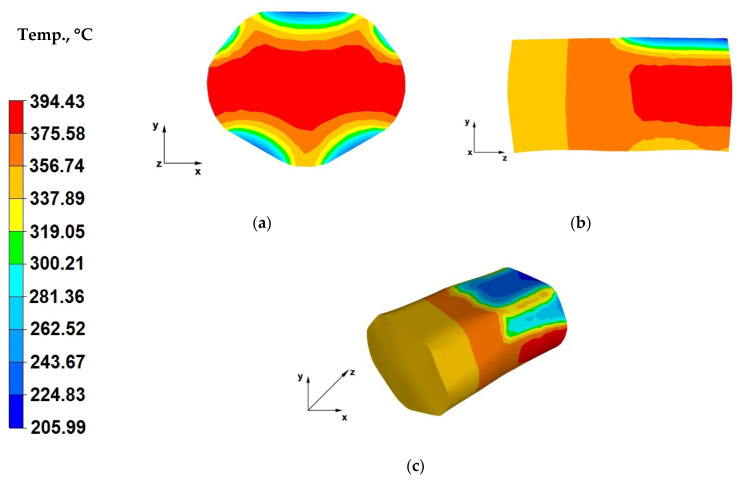
(**a**) Temperature distribution over the cross-section of an AZ91 alloy forging deformed within the second pass, with a relative reduction of 25%, in trapezoid-rhombic dies; (**b**) Temperature distribution over the longitudinal-section of an AZ91 alloy forging deformed within the second pass, with a relative reduction of 25%, in trapezoid-rhombic dies; (**c**) Axonometric view of the temperature distribution over the surface of an AZ91 alloy forged within the second forging pass, with a relative reduction of 25%, in trapezoid-rhombic dies.

**Figure 13:**
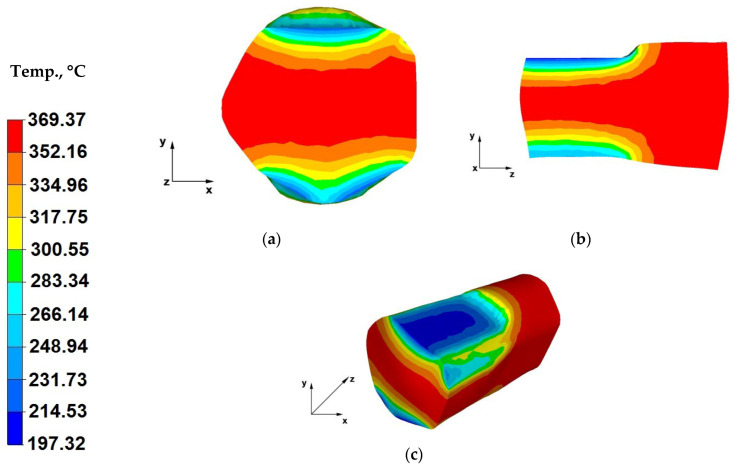
(**a**) Temperature distribution over the cross-section of an AZ91 alloy forging deformed within the third pass, with a relative reduction of 40%, in trapezoid-rhombic dies, after a 90° turning of the forging; (**b**) Temperature distribution over the longitudinal-section of an AZ91 alloy forging deformed within the third pass, with a relative reduction of 40%, in trapezoid-rhombic dies, after a 90° turning of the forging; (**c**) Axonometric view of the temperature distribution over the surface of an AZ91 alloy forged within the third forging pass, with a relative reduction of 40%, in trapezoid-rhombic dies, after a 90° turning of the forging.

**Figure 14:**
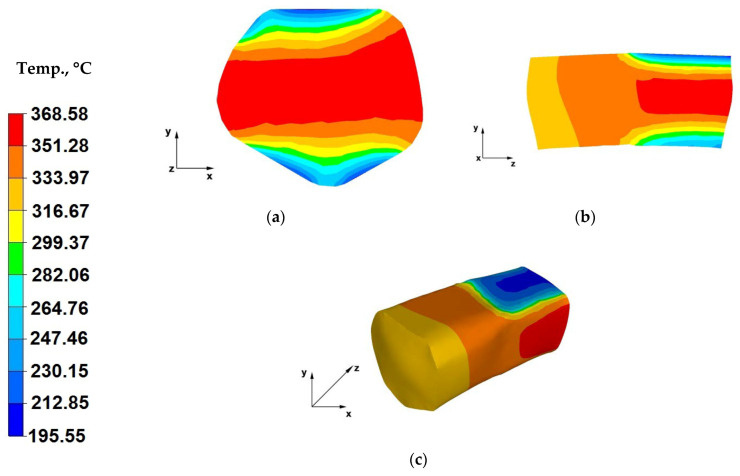
(**a**) Temperature distribution over the cross-section of an AZ91 alloy forging deformed within the fourth pass, with a relative reduction of 40%, in trapezoid-rhombic dies, after a 90° turning of the forging; (**b**) Temperature distribution over the longitudinal-section of an AZ91 alloy forging deformed within the fourth pass, with a relative reduction of 40%, in trapezoid-rhombic dies, after a 90° turning of the forging; (**c**) Axonometric view of the temperature distribution over the surface of an AZ91 alloy forged within the fourth forging pass, with a relative reduction of 40%, in trapezoid-rhombic dies, after a 90° turning of the forging.

**Figure 15:**
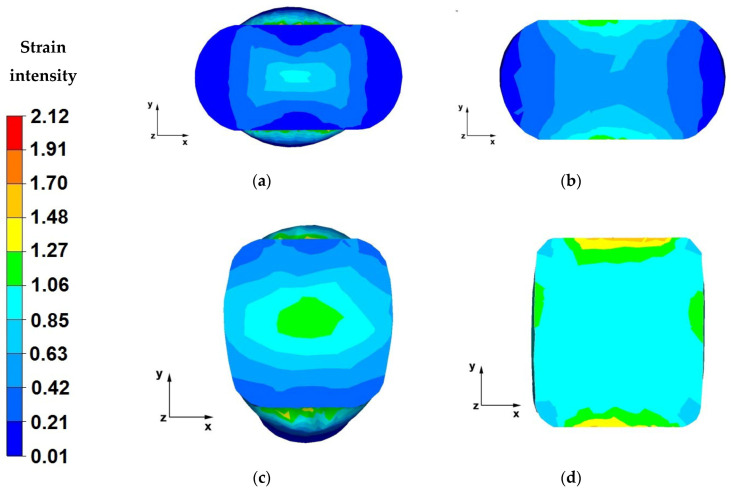
(**a**) Distribution of strain intensities over the cross-section of an AZ91 alloy forged within the first pass, with a relative reduction of 40%, in flat dies; (**b**) Distribution of strain intensities over the cross-section of an AZ91 alloy forged within the third pass, with a relative reduction of 40%, in flat dies; (**c**) Distribution of strain intensities over the cross-section of an AZ91 alloy forged within the fourth pass, with a relative reduction of 40%, in flat dies, after a 90° turning of the forging; (**d**) Distribution of strain intensities over the cross-section of an AZ91 alloy forged within the sixth pass, with a relative reduction of 40%, in flat dies, after a 90° turning of the forging.

**Figure 16:**
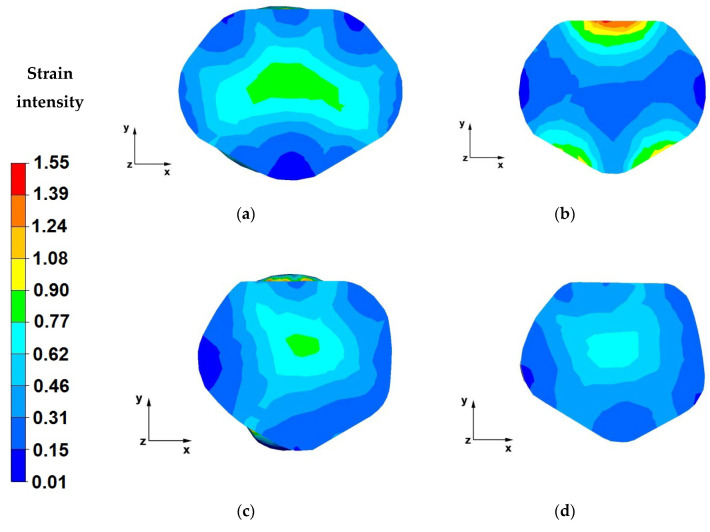
(**a**) Distribution of strain intensities over the cross-section of an AZ91 alloy forged within the first pass, with a relative reduction of 25%, in trapezoid-rhombic dies; (**b**) Distribution of strain intensities over the cross-section of an AZ91 alloy forged within the second pass, with a relative reduction of 25%, in trapezoid-rhombic dies; (**c**) Distribution of strain intensities over the cross-section of an AZ91 alloy forged within the third pass, with a relative reduction of 40%, in trapezoid-rhombic dies, after a 90° turning of the forging; (**d**) Distribution of strain intensities over the cross-section of an AZ91 alloy forged within the fourth pass, with a relative reduction of 40%, in trapezoid-rhombic dies, after a 90° turning of the forging.

The paper analyses the metal strain intensity and temperature distributions within the hot forging process involving AZ91 magnesium alloy bars. The results of temperature distribution from numerical tests are shown in Figure 7, Figure 8, Figure 9, Figure 10, Figure 11, Figure 12, Figure 13 and Figure 14 and the test results for theoretical strain intensity distributions are shown in Figure 15 and Figure 16.

### 7.1. Analysis of the Temperature Changes during the Elongation Process

The objective of the conducted analysis was to determine the temperature changes in a forged alloy, especially in areas adjacent to the areas interfacing with dies. Too high material cooling in the contact zone may cause the appearance of cracks and delaminations of the deformed material. Furthermore, the analysis also included the temperature distribution throughout the volume of the elongated bar, in order to determine the material temperature changes in the course of the forging process. Excessive decrease of the material temperature hinders the plastic flow of the metal within the open die forging processes and contributes to the formation of cracks.

Figure 7a shows a temperature distribution over the cross-section of an AZ91 alloy bar forged within the first pass, with a relative reduction of 40%, in flat dies. The data included in Figure 7a indicates that at the interface between the material with an initial temperature of 400 °C and flat dies with an initial temperature of 25 °C there was a cooling of the forged alloy by 260 °C. This was the consequence of the forged material transferring heat to the flat die material. The central parts of the forged alloy and areas by the free surfaces, free from the impact of dies, experienced a slight initial temperature increase, due to the deformation occurring. During the successive forging passes, the bar cooling processes was slower due to increasing die temperature in contacting areas up to approximately 200 °C, owing to the heat emitted by the forged material. During successive forging passes, especially after 90° turning, the temperature in the surface areas of the forged AZ91 alloy increased due to the deformation occurring and metal flow towards the free surfaces, unrestricted by the impact of flat die working planes.

Figure 7b shows the temperature distribution throughout the bar longitudinal section during the first forging pass. The data therein indicates that there is a significant temperature decrease to 139 °C at the interfaces between the forged material and working surfaces of the dies, while the metal temperature drop in the central areas was relatively low (ingot axis temperature of 323 °C). This is associated with heat transferred from the forged alloy to the die surface, which was also visible in Figure 7a. The material flows freely towards the Z and X axes in the central forging sections. The deformation occurring in these direction results in maintaining the set initial temperature of the forged alloy, i.e., 400 °C. The entire model process is aimed at reflecting the actual AZ91 alloy forging process, which is why the initial die temperature was set at 25 °C. The dies are not heated during the true forging process.

Figure 7c shows the temperature distribution on the forged bar surface, during the first straining stage, which indicates that the temperature drop on the contract surface between the metal and tools is uneven. The highest temperature drop was observed in the central part of the contact area.

Figure 8a shows the temperature distribution throughout the cross-section of a bar forged within the third forging pass, prior to turning the forging by 90°. The shown data indicates that there was a metal temperature drop to about 155 °C in the zones adjacent to the contact surface with tools. The forged bar surface temperature difference between the first and third forging passes was approximately 30 °C (Figure 7a). This results from the heat being given away by the forged batch to the environment during individual forging passes, as well as the heating up of the die working surfaces. The temperature of the forged bar in its central section is lower relative to the bar temperature in the first forging pass. Near the axis of the forged bar and in the areas unrestricted with the working surfaces of flat dies, the material flows freely and, despite the large straining action, induced by the applied relative reduction as high as 40%, the temperature dropped slightly from 400 °C to 391 °C. This is associated with heat emitted to the environment during the long elongation operation (hydraulic press slide feed rate of 8 mm/s) in successive forging passes. It is worth noting that between successive die impacts, the batch is shifted in order to apply subsequent strikes and, as a result, this requires time for lifting the dies and lowering them again onto the surface of the metal (forging pass executions). In addition, please note that the reference tests are conducted for strain conditions characterizing a hydraulic press, where the breaks between the impacts are significantly longer than in the case of a hammer.

The data included in Figure 8b indicates that the temperature in the metal zones adjacent to the material-tool contact surface, strained after the first and second forging passes increased by about 200 °C. This is a positive result, since the hot forging operation is always aimed at achieving the lowest possible metal temperature difference, between the start and end of the process. This temperature increase results from the lack of die action in these zones, as well as straining action with applied relative reduction of 40%. Furthermore, heat from the heated alloy zone with the highest temperature (370 °C) flows to cooler zones, cooled by dies in contact with them. In the axial section of the forged alloy impacted by dies, the metal temperature is equal to the initial batch temperature due to the highest straining action.

Figure 8c shows the temperature distribution throughout the surface of a bar forged within a third forging pass. The data included in this figure indicates that the temperature of the forged bar in the first and second forging passes increased to almost the initial temperature and was from 344 to 368 °C (the highest drop amounted to 56 °C). Hence, the metal temperature drop is slight, despite the fact that the forging operation was conducted using a hydraulic press, with a slide feed rate of 8 mm/s and this was already the final forging stage, prior to turning the forging by 90°. Such a slight temperature drop was caused by the plastic strain thermal effect due to applying relative reductions of 40% within the three forging passes.

Figure 9a shows the temperature distribution throughout the cross-section of a bar forged within the fourth forging pass, after rotating the forging by 90 degrees, that the alloy temperature in areas adjacent to the contact surface with the dies was approximately 190 °C, which was caused by increased die temperature during previous forging operations. The die working surface temperature after the third forging pass increased to about 150 °C. This resulted in the fourth pass die temperature drop in the axial areas of the bar not being so significant and ranging from 361 to 364 °C, while the forging temperature in the intermediate zones between the axes and the surfaces was lower and varies from 309 to 327 °C.

Figure 9b shows the temperature distribution throughout the longitudinal section of a bar forged within the fourth forging pass. Based on the analysis of the data in Figure 9b, it can be concluded that the material temperature, over the majority of its volume, owing to the intensive plastic strain translating directly into applied relative reductions of 40%, is satisfactory and close to the initial batch temperature varying in the range of 344–361 °C. Slight material cooling in the axial zone of the forging, towards the Z-axis can be observed (freely flowing material). Whereas the material temperature in the die impact zone was 361 °C.

Figure 9c shows the temperature distribution throughout the surface of a bar forged within the fourth forging pass, after turning the forging by 90°. The data in Figure 9c indicates that the forging temperature distribution is uniform, except for areas near the contact surface with the dies. The temperature drop resulted from the process of emitting material heat to the environment. The alloy temperature is relatively high in most zones of the strained material, owing to the continuous plastic strain action, applied by dies pressing the material in successive forging passes. The material temperature in the zones adjacent to the surfaces of contact with the dies is also increasing as a consequence of increasing die temperature, which is approaching the temperature of the forged alloy.

Analysis of the data in Figure 10a indicates that in the case of the sixth pass, the cooling down of the metal areas within the contact zones with dies was lower and the forging temperature in these areas was 191 °C. Axial forging zones recorded a temperature drop of 53 °C, from the initial temperature of 400 °C down to 347 °C. Please note that this is the last forging pass and the metal temperature in the final forging operation is relatively high and amounts to 347 °C. This evidences that the AZ91 magnesium alloy accumulates heat really well during the flat die forging operation. Heat transfer from the forging to the environment is slow. The heat emission from steel die blocks also dropped significantly because the analysis of the forging numerical modelling indicates that, during the process, the dies heated up from the ambient temperature of 25 to 170 °C during the sixth forging pass. This proves that forging in flat dies with a high relative reduction of 40%, without reheating the dies, should not create problems involving excessive cooling of the strained bar due to heat exchange with the environment and die material, in the course of forging a bar with a diameter of φ 80 mm and length *l* = 80 mm.

The data in Figure 10b indicates that the temperature on the surface of the forging cross-section ensures good conditions for conducting the forging operation (material is still plastic), since the entire zone of the forged alloy, beyond the die impact area, exhibits a temperature of 332 °C. The temperature increases up to 347 °C in the central part of the bar is cause by strain action caused by the maximum forging reduction applied during the forging operation.

When analysing the temperature field distribution throughout the surface of a forged forging (Figure 10c), it can be concluded that the entire forging operation was conducted at an optimal alloy temperature, which can be seen on both the cross- and longitudinal-sections (Figure 10a,b), as well as the whole surface of the reforged bar, except for the zones adjacent to the material-dies contact surface. Applying high relative reductions (40%) is advantageous for the forging process itself, as well as maintaining proper temperature-related conditions of the forging forming process, hence providing it with the assumed shape and external dimensions. Owing to the high action of the plastic strain, the material does not cool down below the temperature required by good forging practice for conducting hot forging of bars.

The data in Figure 11a indicates that cooling of the forging zones adjacent to the working surfaces of shaped dies was lower compared to the temperature drops of the alloy strained using flat dies (Figure 7a). A metal temperature drops down to 169 °C was recorded, despite the fact that also in the case of this operation the initial die temperature was 25 °C, similar to forging in flat dies. This was caused by a shorter contact time between the bar and die surfaces, due to the forging reduction of 25% applied in this case. The application of lower relative reductions led to shortened duration of the pressure applied on the forging, relative to the straining with a relative reduction of 40%.

In the initial forging operations, prior to rotating the forging by 90°, as a result of using trapezoid-rhombic dies, it was impossible to introduce higher forging reductions for bars with a diameter φ = 80 mm, because the hammer block would already be in contact with the lower die. This is associated with their geometry and dimensions, compared to flat dies, where there are only two parallel working surfaces. In the case of trapezoid-rhombic dies, the material-tools contact surface expands, the forging free surface is significantly smaller and the dies also impact the lateral surfaces of the strained bar (Figure 11a). Only after turning of the forging (material freely flowing along the X-direction) does the bar achieve sufficient height for applying forging reductions above 25%. Based on the analysis of the data in Figure 11a, it can also be concluded that the material in trapezoid-rhombic dies is strained not only in the Y-direction, but also at appropriate angles, which is why we can observe cooling down of not only the upper and lower zones of the forged material but also of the lateral ones. The temperature in these areas amounted to 300 °C.

When analysing the data in Figure 11b, it can be concluded that both in the case of the cross-section, as well as the longitudinal-section, the bottom section of the forging experiences a lower metal temperature drop, because the flowing plastic material failed to fully fill the top zones of the bottom rhombic die. Whereas in the upper section of the forging, the strained material fully filled the working area of the upper trapezoid die, transferring heat to the die block, which resulted in decreased metal temperature in this area, from the initial temperature of 400 °C to a temperature in the range from 214 to 169 °C.

Figure 11c shows the temperature distribution on the surface of a bar forged within the first strain stage. A temperature drop on the surface of the upper contact zone between the bar and the working surface of the trapezoid upper die can be seen, which occurred due to heat conducted from the bar to the die, with a temperature of 25 °C. A temperature drop by the material-tools contact surfaces was also observed in the bottom section of the bar.

Figure 12a shows the temperature distribution throughout the bar longitudinal section during the second forging pass. The forging batch heated up to 400 °C, in its strain crucible, through the entire surface of contact with the tools, releases heat to the steel die blocks, which heat up faster than in the case of flat-die forging. This is why, a lower metal temperature drop (down to 205 °C) was observed by the surfaces of contact with the tools, which means that the dies heated up from the forged bar, during the previous forging pass. For comparison, the bar surface near the contact surface with the dies cooled down to 169 °C in the first pass (Figure 11a). A metal temperature drop to 155 °C was recorded in the case of flat-die forging, in the first stage of forging forming (Figure 8a). Despite the lower strain action and friction force values, the temperature of the bar shaped in two first forging passes is sufficient for rational straining and free flow of the material towards the surfaces unrestricted by the dies.

The forging in trapezoid-rhombic dies, due to the restricted free flow of the material caused by die shape, can be compared to the die forging process.

The analysis of the data in Figure 12b indicates that throughout almost the entire volume of the forged bar, its temperature stayed at a satisfactory level (range of 337–394 °C), which was caused by the slow release of heat to the environment. In the case of forging a bar in trapezoid-rhombic dies, the distribution of pressure force and friction force impact vectors is different than in the case of flat die forging. Despite applying almost halved forging reductions, owing to the specific impact of trapezoid-rhombic dies on the formed material, the strain crucible experiences intersecting pressure and friction force impact vectors, which results in increasing tangential stress values, translating to growing strain action values. The temperature of the strained material increases with increasing value of strain action.

The data in Figure 12c indicates that the temperature of the bar in zones outside of the strain crucible ranged from 356 to 375 °C, whereas in the strain crucible it amounted to approximately 394 °C. This is caused by plastic strain, which induces material flow in the direction of free surfaces, located outside of the tool impact range. The increased plastic strain action influences maintaining the temperature of the strained material. Hot-forging of bars in trapezoid-rhombic dies is advantageous owing to maintained proper temperature of the strained alloy and its ensured good plasticity. Such a forging method provides rational forming of the forging, with a simultaneously low demand for the forces required for deforming individual forging zones. No significant temperature, relative to the first forging pass, was observed on the material-tools contact surfaces (contact surface temperature of 224 °C).

Based on the analysis of temperature distributions presented in Figure 13a, it can be concluded that the axial zones of the forging recorded a temperature drop of approximately 30 °C. This is caused by a longer break in the forging process, because apart from the time needed for moving the hammer block, the forged bar had also to be turned by 90° and moved away from the strain crucible, and then the hammer block had to be lowered in order to restrain the forging. During this period, the working surfaces of the dies emitted heat to the environment and the material temperature on the contact surface with the tools slightly decreased. The minor drop of the bar temperature did not lead to noticeably decreased material plasticity.

The data in Figure 13b indicates that the temperature of the axial zone of the forging strain crucible is 369 °C. The farther from the forging centre, the higher the temperature drop in the strained alloy. The material temperature on the tool contact surface is 197 °C.

Figure 13c shows the temperature distribution on the surface of a bar forged within the third strain stage. The minor temperature drop of the strained material, despite the interoperational gap associated with turning of the forging, resulted from the applied relative reduction of 40%.

Based on the data included in Figure 14a, it can be stated that the relative reduction of 40% applied in the fourth (ultimate) pass did not cause an increase of the material temperature, despite the shorter process break. The process break was caused by drawing back the hammer block, moving the forging and refixing the die. The temperature of the strained bar stayed within the optimal temperature ranges, required for the forging operation.

The data in Figure 14b indicates that the metal temperature in the axial zone of the forging, located in the strain crucible, ranged from 316 to 369 °C. The larger the distance from the centre of the forging, the greater the metal temperature drop. The temperature at the material-tools interface was 195 °C. The temperature distribution within the forged bar achieved in this case was the same as for the third forging pass.

Based on the analysis of the data in Figure 14c, it can be concluded that the engineered forging process managed to maintain an optimal temperature regime in the forging, during the forging operation, which ensures rational material flow in the strain crucible, hence, rational strain of the stretched forging. The forging temperature decreased by about 85 °C in the course of the process. During individual forging passes, the applied forging reductions initiated strain action in the forging-tool contact zones, thus contributing to increasing temperature of the material in the zones of contact with the tools. This indicates that the analysed shaped die forging operation maintained appropriate temperature within the strained bar, which enabled free, plastic forming of the material.

### 7.2. Analysis of the Strain Intensity Distribution during Forging Process

Figure 15 show strain intensity distributions achieved during the numerical forging of the AZ91 magnesium alloys in flat dies.

The data in Figure 15a indicates that the strain intensity value within the axial zone of the forging varied from 0.63 to 1.06, while ranging from 0.21 to 0.42 in the die action zones and the zones of material free flow along the X-axis. Such a distribution is closely associated with the impact of flat dies, which induce specific flow kinematics for the strained material. The material in the axial zone of the bar, along the Z-axis, was forged evenly and contains strains of the lowest value, which is of significance in the case of bar elongation, because through-penetration forging of the material in the forging core is the most important issue, which is not always successful in forging operations. Obviously, the metal zones under the impact of dies and the zones of material free flow along the X-axis were not sufficiently forged and we cannot speak of strain uniformity throughout the entire section of the forged bar. The most important thing within the first stages of the forging hot forming is to conduct the process, so that the metal in the axial zones of the forging, most remote from the direct impact of the dies, experiences large strains. If a significant drop in the semifinished product temperature is experienced in the course of forging, due to heat being dissipated to the environment and dies, it is hard to achieve correct forging of material zones, because of lowered plastic flow of material particles and higher strain resistance.

Based on the data in Figure 15b, it can be concluded that the strain intensity value in the central zone of the material subject to a third forging pass ranges from 0.6 to 1.06, while in the case of zones near the contact surface with the die, they varied from 1.07 to 1.6, and from 0.002 to 0.42 in the zones of material free flow along the X-axis. The data analysis indicates that there was a uniform strain intensity distribution in the strain crucible. This proves the possibility of achieving homogeneous mechanical properties of a finished product. When hot and cold forging products, the objective is to ensure uniform distribution of the mechanical properties throughout the volume of a finished product. This influences the good quality and reliability of such a product.

The data in Figure 15c indicates that after turning the forging by 90° and applying another forging reduction in previously widened spots, the material moves along the Z-axis and the strain intensity value varies from 1.06 to 1.27. Previously unstrained zones, after bar turning found themselves under direct action from the dies and the strain intensity value therein is 0.63. The strain intensity distribution throughout the cross-section of the forging is more uniform, which is particularly important with regard to obtaining homogeneous mechanical properties within the entire forged bar.

Based on the data in Figure 15d, one can conclude that after the last forging pass, which completes the flat die forging operation, uniform strain intensity (1.06) was achieved throughout the cross-section of the bar. Areas with other strain intensity values are found near the contact surface with the dies. The strain intensity values therein range from 1.27 to 1.48. In the case of the analysed forging operation, the nature of strain distribution is satisfactory, which means that uniform strain distribution can be achieved in the studied alloy strained in flat dies. This can constitute a base to conclude that the mechanical properties of a finished product will be uniform throughout its volume. Selecting a relative reduction of 40%, relative feed of 0.8 and a rotation angle of 90° were the right choice.

Figure 16 show strain intensity distributions achieved during the numerical forging of the AZ91 magnesium alloys in trapezoid-rhombic dies.

The data in Figure 16a indicates that the strain intensity values were the highest in the axial zone of the forging, along axis-Z, and fell within a range of 0.29–0.35. The strain intensity distribution within the analysed area was uniform. The strain intensity values in the areas of direct die action on the alloys were also uniform and fell within a range of 0.17–0.23. Based on the analysis of the entire forging cross-section, it is possible to conclude that the intensity distribution is more uniform relative to the distribution for forging in flat dies (Figure 15a).

The analysis of the data in Figure 16b indicates that for the second forging pass, in the axial zone of the forging, the strain intensity values ranged from 0.21 to 0.28. In the material-tool contact zone, the strain intensity value fell in the range of 0.35–0.49. Strain intensity distribution is uniform, similar to the previous forging pass.

The data included in Figure 16c indicates that the strain intensity value within the axial zone of the forging was the highest and amounted to 0.42. The pressure force vectors originating from the action of both dies cross in the forged bar axis, hence the strain accumulation in this zone. Larger forged bar (compared to the elongation in the first and second passes) was observed in this pass. The reason behind the larger elongation was the shape of the rhombic and trapezoidal die working surfaces, limiting the material flow along the X and Y axes. It is an intended effect achieved through designing such a die shape. Reforge uniform throughout the volume of the material is critically important due to the requirement of homogeneous mechanical properties of the final product.

The analysis of the data shown in Figure 16d leads to a conclusion that the strain intensity distribution after the final forging pass was still uniform.

## 8. Final Conclusions

The following final conclusions were drawn based on analysing the results of conducted tests:

- In economic and process terms, it is rational to conduct AZ91 magnesium alloy bar elongation operations in trapezoid-rhombic dies (designed by the authors), since it leads to reducing the number of forging passes from six in the case of flat dies, to four in the case of shaped dies. A reduced number of forging passes does not influence the end dimension of a finished product.
- The application of shaped dies for elongation operations, compared to conducting the process in flat dies, leads to obtaining a more uniform temperature distribution between the central forging part and the alloy areas by the material-tool contact surfaces, which impact a better plastic flow of the material.
- When conducting elongation operations in both flat, as well as shaped dies, there was a significant drop in the bar temperature in areas adjacent to the material-tool contact surface. The cause behind this decrease was transferring heat to unheated dies. The forging areas near the action zones of dies, after cooling and further heat treatment are treated as machining losses.
- A very uniform distribution of the strain intensity value can be found throughout the entire bar during elongation in trapezoid-rhombic dies. However, strain intensity values are lower compared to the values obtained when elongation in flat dies. Higher strain intensity value proves good material processing, which results in obtaining better mechanical properties for the finished product.

The following assumptions for the forging technology were suggested when analysing the AZ91 alloy bar elongation in flat and trapezoid-rhombic dies:

- trapezoid-rhombic dies should be used for the initial forging forming stages, since they can provide a uniform distribution of strain intensity,
- it seems justified to use flat dies for the ultimate forging forming stages, since they provide higher strain intensity values within the volume of the strained bar,
- a forging reduction of 40% shall be applied for the initial forging forming stages, due to the shape of used dies,
- a forging reduction of 40% can be applied in the final stages,
- a relative feed of 0.8 shall be applied for the elongation operations,
- the initial bar temperature assumed for the numerical simulations proved correct, since there was no significant material cooling observed in the course of the elongation operation, which could result in decreased flow stress of the strained alloy,
- when elongation using both flat, as well as trapezoid-rhombic dies, apply forging turning of 90°.

## Figures and Tables

**Table 1:** Chemical composition of the investigated alloy [%].

Alloy	Zn	Al	Si	Cu	Mn	Fe	Ni	Mg
AZ91	0.59	8.98	0.05	0.006	0.23	0.013	0.003	R

**Table 2:** Parameter values obtained from the approximation of Equation (1).

	A	m_1_	m_2_	m_3_	m_4_	m_5_	m_7_	m_8_	m_9_
**AZ91**	5985.99	−0.0116	0.37027	0.20062	−1.948 × 10^−5^	−0.008	0.5807	0.00021	2.54972
